# Kupffer Cell Inactivation Alters Endothelial Cell Adhesion Molecules in Cecal Ligation and Puncture-Induced Sepsis

**DOI:** 10.3390/biom14010084

**Published:** 2024-01-09

**Authors:** Sumeet Manandhar, Ravinder Reddy Gaddam, Stephen Chambers, Madhav Bhatia

**Affiliations:** Department of Pathology and Biomedical Science, University of Otago, Christchurch 8140, New Zealand; sumeet.manandhar@postgrad.otago.ac.nz (S.M.); ravinderreddy-gaddam@uiowa.edu (R.R.G.); steve.chambers@otago.ac.nz (S.C.)

**Keywords:** gadolinium chloride, sepsis, adhesion molecules, endothelial cells

## Abstract

The activation of Kupffer cells, resident macrophages in the liver, is closely associated with the inflammatory response during sepsis, which leads to multiple-organ failure. However, how Kupffer cell activation affects adhesion molecules (ICAM-1 and VCAM-1) in sepsis has not been determined. This study investigated Kupffer cell inactivation’s (by gadolinium chloride; GdCl_3_) effects on adhesion molecule expression in CLP-induced sepsis. The induction of sepsis resulted in increased expression of liver and lung ICAM-1 and VCAM-1. GdCl_3_ pretreatment significantly decreased liver ICAM-1 expression but had no effect on VCAM-1 expression. In contrast, GdCl_3_ pretreatment had no effect on sepsis-induced increased adhesion molecule expression in the lungs. Similarly, the immunoreactivity of ICAM-1 was decreased in liver sinusoidal endothelial cells but increased in pulmonary endothelial cells in septic mice pretreated with GdCl_3_. Further, GdCl_3_ pretreatment had no effect on the immunoreactivity of VCAM-1 in endothelial cells of the liver and lungs. Hence, the findings of this study demonstrate the differential effects of Kupffer cell inactivation on liver and lung adhesion molecules and suggest the complexity of their involvement in the pathophysiology of sepsis.

## 1. Introduction

Kupffer cells are resident liver macrophages that regulate the metabolic activity of the liver [1]. During sepsis, the liver has a major function in removing bacteria and mediating the inflammatory response. Kupffer cells are responsible for sequestering bacteria and toxins from the gastrointestinal tract, which are delivered to the liver through the portal vein [2]. Kupffer cells release various products upon activation: cytokines (TNF-α, IL-1β, and IL-6), chemokines (MIP-2α and MCP-1), eicosanoids, nitrogen radicals, and reactive oxygen species.

Numerous investigations have demonstrated that the interaction of Kupffer cells with endotoxins is the initial event during endotoxemia/sepsis. This causes liver injury, extrahepatic organ failure, and systemic inflammation [3,4,5]. In addition, Kupffer cells contribute to the inflammatory response through the excessive production of cytokines and chemokines during sepsis. However, the role of Kupffer cells extends beyond cytokine and chemokine production. Understanding their involvement in the expression of adhesion molecules in tissues would contribute to the knowledge on the role of Kupffer cells in the pathophysiology of sepsis and septic shock. GdCl_3_ has been demonstrated to block Kupffer cells’ phagocytic activity effectively, providing a useful strategy to investigate their contribution to the pathophysiology of endotoxemia and sepsis [5,6,7,8].

Many studies have demonstrated that liver sinusoidal endothelial cells in the liver and pulmonary endothelial cells in the lungs play a key role in leukocyte recruitment [9,10]. Lymphatic vessel endothelial hyaluronan receptor-1 (LYVE-1) has been identified as a marker of liver sinusoidal endothelial cells, and CD31 has been shown to be a marker of pulmonary endothelial cells. Hepatic injury and lung injury are significantly influenced by endothelial–leukocyte interactions [11,12]. These interactions are facilitated via cellar adhesion molecules such as Intercellular Adhesion Molceulse-1 (ICAM-1) and Vascular cell adhesion molecule-1 (VCAM-1) present on the surface of endothelial cells, resulting in the infiltration of leukocytes to the site of infection [13,14,15,16,17,18]. Pro-inflammatory cytokines, mainly TNF-α, upregulate the expression of both ICAM-1 and VCAM-1 [19,20,21], and these adhesion molecules are highly expressed in endothelial cells during sepsis.

In vitro studies have demonstrated that lipopolysaccharide (LPS) stimulates Kupffer cells to express TNF-α, resulting in markedly increased expression of ICAM-1 on liver sinusoidal endothelial cells (LSECs). Furthermore, treatment with dexamethasone (an anti-inflammatory drug) inhibited the production of TNF-α by LPS-stimulated Kupffer cells. Subsequently, it reduced the expression of ICAM-1 and the adhesion of neutrophils on LSECs [22]. A study from our group has shown that sepsis disrupts LSECs, resulting in defenestration (fenestrae are reduced in number, resulting in the sinusoid appearing more like an ordinary capillary) and the formation of gaps (large defects in the LSECs) that led to liver injury. Pretreatment with GdCl_3_ decreased defenestration and TNF-α expression during sepsis, providing protection against liver injury [23].

In patients with sepsis, acute lung injury (ALI) and acute respiratory distress syndrome (ARDS) are the precursors to the progression of multiple-organ failure [24]. During ARDS, ICAM-1 and VCAM-1 expression on pulmonary endothelial cells increased [25]. Despite significant efforts to understand the lung injury caused by endotoxemia, sepsis, and the effects of GdCl_3_ pretreatment, the impact of GdCl_3_ on lung injury remains unclear, although several important results have been reported. Pretreatment with GdCl_3_ has been demonstrated to prevent ozone-induced pulmonary injury by altering the production of pro-inflammatory mediators [26]. GdCl_3_ pretreatment has also decreased the activation of caspase-3, resulting in reduced LPS-induced pulmonary apoptosis. However, it has been observed that GdCl_3_ treatment in lungs did not reduce elevated mRNA expression of pro-inflammatory cytokines, such as TNF-α, IL-6, and IL-1β [27]. Additionally, pretreatment with GdCl_3_ in CLP-induced sepsis significantly increased the expression of various cytokines, including TNF-α, and increased the MPO activity and infiltration of leukocytes in the lung tissue [23]. The disparity in Kupffer cells’ role in lung inflammation across different disease models of endotoxemia and sepsis suggests that more work is needed to clearly understand the effect of Kupffer cells on the lungs during sepsis.

This study aimed to explore the effects of Kupffer cell inactivation on ICAM-1 and VCAM-1 expression in liver and lung endothelial cells in mice following CLP-induced sepsis. This study tested the hypothesis that Kupffer cells contribute to tissue injury in sepsis via the alteration of adhesion molecules (ICAM-1 and VCAM-1) present on the endothelial cells in liver and lung tissue.

## 2. Materials and Methods

### 2.1. Induction of Polymicrobial Sepsis in Mice

Thirty-two (C57BL6 mice, male, 25–30 g) mice were allocated to control (sham and sham pretreated with GdCl_3_) or investigational (CLP and CLP pretreated with GdCl_3_) groups in a randomized manner, with each group containing eight mice (*n* = 8). Before CLP and sham operations, mice were pretreated with 10 mg/kg of GdCl_3_ (i.v.) through the tail vein, while saline-treated sham and CLP mice served as vehicle-treated controls. All animal procedures were approved by the University of Otago Animal Welfare Office and Ethics Committee.

Before the surgical procedures, inhaled isoflurane (2%, 1 L/min O_2_) was used to anesthetize the mice. Sepsis was conducted by CLP with minor changes to a previously documented protocol [28]. Briefly, a small incision along the midline of the anterior abdomen was made to expose the cecum. Then, the cecum was ligated using 5.0 Silkam thread, positioned 8–10 mm from its end, without impeding the bowel passage. Subsequently, the cecum was perforated at two different locations with a 22-gauge (22G) needle, allowing the extrusion of a small amount of feces from each hole. The cecum was repositioned, and the abdominal was sutured back. The sham control group went through a similar surgical procedure without bowel perforation. A subcutaneous injection of buprenorphine (Temgesic, Indivior Pty Ltd., Sydney, NSW, Australia, 0.2 mg/kg) for analgesia was given 45 min preoperatively and 3 h postoperatively. Eight hours post-surgery, mice were euthanized through IP injection of pentobarbital sodium (150 mg/kg). Liver and lung tissue sections were fixed in 10% formalin for over 24 h and then embedded in paraffin wax. The remaining tissue samples were stored at −80 °C for quantification of adhesion molecules.

### 2.2. Enzyme-Linked Immunosorbent Assay

Adhesion molecules’ concentrations (ICAM-1 and VCAM-1) in liver and lung tissues were quantified by employing DuoSet ELISA kits from R&D Systems (Minneapolis, MN, USA), following the manufacturer’s guidelines. In total, 20 mg of tissue was homogenized in 1 mL of sodium phosphate buffer (20 mM, pH 7.4) to prepare tissue homogenates. Subsequently, the homogenates underwent centrifugation at 15,000× *g* for 15 min at 4 °C, and the resulting supernatant was utilized for measurement of adhesion molecule concentration. Measurements were normalized based on the protein levels of the tissue samples, as determined using the Bradford protein assay, and the results are expressed as picograms per milligram of protein.

### 2.3. Double Immunofluorescence Staining of Paraffin Sections

Tissues were harvested and immersed in 4% buffered paraformaldehyde for fixation. The fixed tissues underwent processing and were embedded in paraffin. A LEICA microtome (HistoCore BIOCUT R, Leica Biosystems, Mt Waverley, Australia) was used to cut 4 μm thick sections from paraffin-embedded tissues and fixed to a charged glass slide. Following deparaffinization and hydration, antigen retrieval was performed by subjecting slides to a pressure cooker in Tris EDTA pH 9.0 buffer at 100 °C for 4 min. Slides were then cooled for 2 h in the buffer, rinsed in water three times, washed in PBS for 5 min, and permeabilized with PBS-Tween (0.1%) for 15 min. The slides were again washed with PBS three times. A hydrophobic barrier was drawn around the tissue section using an ImmEdge pen (H-4000, Newark, CA, USA). Normal donkey serum (10%) was used to block the sections at room temperature (in a humidifying chamber) for 1 h. The sections were then incubated in primary antibody overnight at 4 °C (in a humidifying chamber) (Table 1). After washing with PBS, the sections were incubated with the relevant FITC- and Cy5-conjugated secondary antibodies (Table 1) for 2 h with DAPI (4′,6-diamidino-2-phenylindole) at room temperature. The slides were rewashed with PBS. Sections were mounted with anti-fading solution and imaged on a Zeiss AxioImager Z1, AxioCamHRc (Carl Zeiss, Waltham, MA, USA).

The mean fluorescence intensity (MFI) of target protein (ICAM-1 and VCAM-1) was determined by an automated region of interest selection method based on signal threshold criteria via Fiji ImageJ–1.54f (downloaded at https://fiji.sc/ accessed on 1 June 2023) according to the protocol previously described [28].

### 2.4. Statistical Analysis

The data are expressed as mean ± SD and were assessed for Gaussian or normal distribution using the Shapiro–Wilk test. For normally distributed data, statistical comparisons were performed using one-way ANOVA with post hoc Tukey’s test. If the data were of non-normal distribution, the Kruskal–Wallis test, a non-parametric method, was applied. All statistical analysis were conducted using GraphPad Prism software (version 9, San Diego, CA, USA), and a statistical significance of *p* < 0.05 was considered.

## 3. Results

### 3.1. Effect of Kupffer Cell Inactivation on Adhesion Molecule Expression in the Liver and Lungs Following Cecal Ligation and Puncture (CLP)-Induced Sepsis

**Figure 1:**
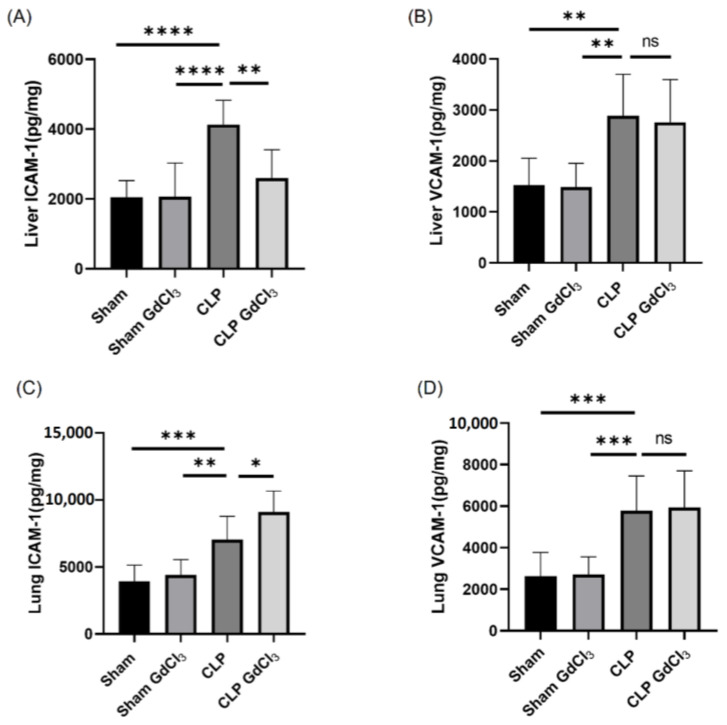
Effect of GdCl_3_ treatment on liver and lung adhesion molecule expression in CLP-induced septic mice. (**A**) Liver ICAM-1, (**B**) liver VCAM-1, (**C**) lung ICAM-1, and (**D**) lung VCAM-1. Results are expressed in pg/mg of protein. All the data are represented as mean ± SD (*n* = 8). One-way ANOVA with post hoc Tukey test was employed. Statistical significance was determined at * *p* < 0.05; ** *p* < 0.01; *** *p* < 0.001; **** *p* < 0.0001; and ns, not significant.

The mean concentrations of the adhesion molecules ICAM-1 and VCAM-1 were significantly elevated in the liver and lungs of CLP-induced septic mice compared to the sham control group. GdCl_3_ pretreatment in mice with CLP-induced sepsis attenuated the increase in ICAM-1 concentration in the liver. In contrast, the concentration of ICAM-1 further increased in the lungs of GdCl_3_-treated septic mice compared to septic mice without GdCl_3_ treatment. However, no significant alterations were observed in the VCAM-1 levels in the liver and lungs of septic mice pretreated with GdCl_3_ compared to those without GdCl_3_ pretreatment (Figure 1).

### 3.2. Effect of Kupffer Cell Inactivation on Immunoreactivity of Liver ICAM-1 and VCAM-1 Co-Localized with Liver Sinusoidal Endothelial Cells

**Figure 2:**
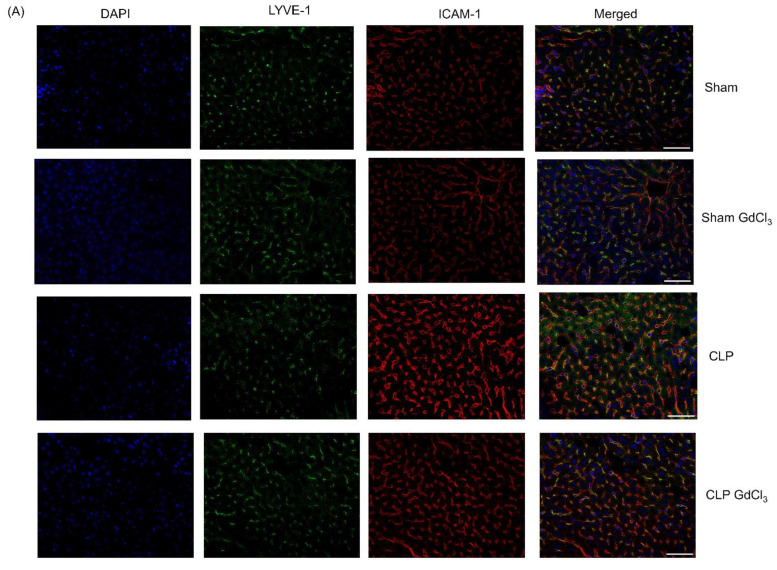

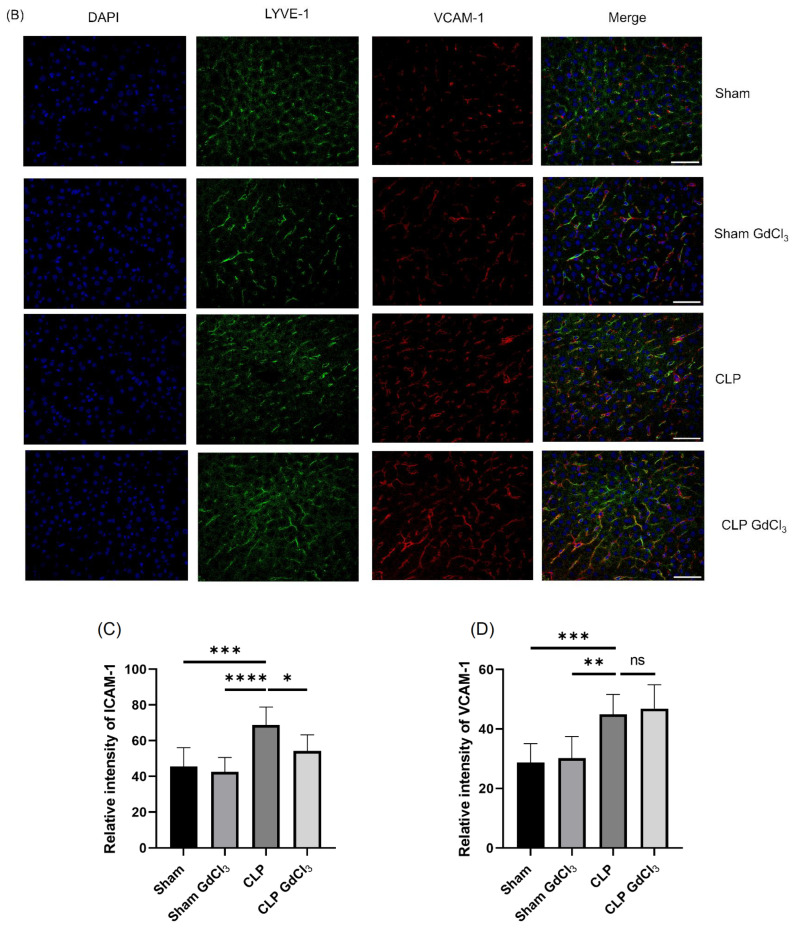
Effect of GdCl_3_ treatment on the immunoreactivity of adhesion molecules on the liver sinusoidal endothelial cells in sepsis. Scale bar: 50 μm. Representative images of ICAM-1 (**A**) and VCAM-1 (**B**) immunoreactivity (Cy3—red) co-localized with LYVE-1 (Alexa Fluor 488—green) after 8 h of CLP or sham operation. DAPI staining was used for the visualization of the nucleus. Semi-quantitative analysis of ICAM-1 (**C**) and VCAM-1 (**D**) immunoreactivity on LSECs were increased in CLP-induced mice compared to sham-operated control mice. Results were expressed in relative fold increase. All data are represented as mean ± SD (*n* = 8). One-way ANOVA with post hoc Tukey test was employed. Statistical significance was determined at * *p* < 0.05; ** *p* < 0.01; *** *p* < 0.001; **** *p* < 0.0001; and ns, not significant.

Increased ICAM-1 (Figure 2A–C) and VCAM-1 (Figure 2B–D) immunoreactivity was observed in LYVE-1 compared to the sham control group. After treatment with GdCl_3_ in the CLP-induced septic mice, ICAM-1 immunoreactivity intensity was significantly reduced in structures identified with LYVE-1 staining compared to CLP-induced septic mice without GdCl_3_ treatment. However, there was no alteration in the VCAM-1 immunoreactivity intensity in structures identified with LYVE-1 staining in septic mice pretreated with GdCl_3_ compared to septic mice without GdCl_3_ treatment.

### 3.3. Effect of Kupffer Cell Inactivation on Immunoreactivity of Lung ICAM-1 and VCAM-1 Co-Localized with Pulmonary Endothelial Cells

**Figure 3:**
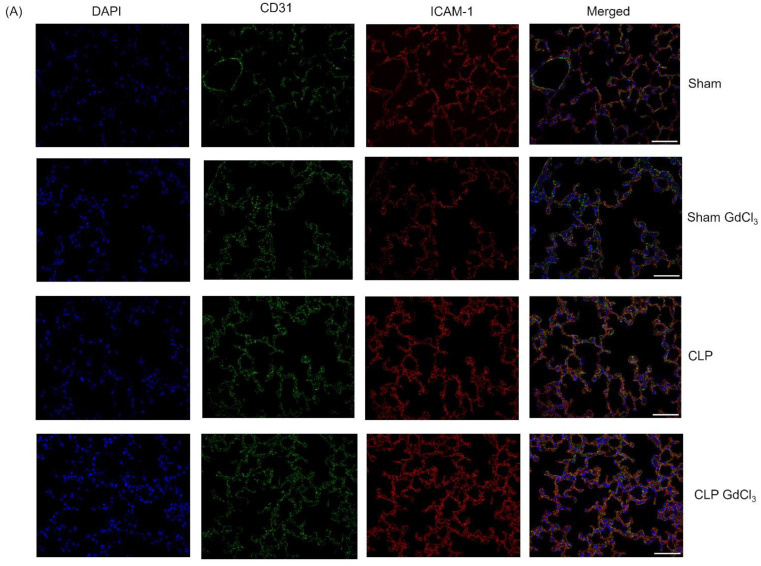

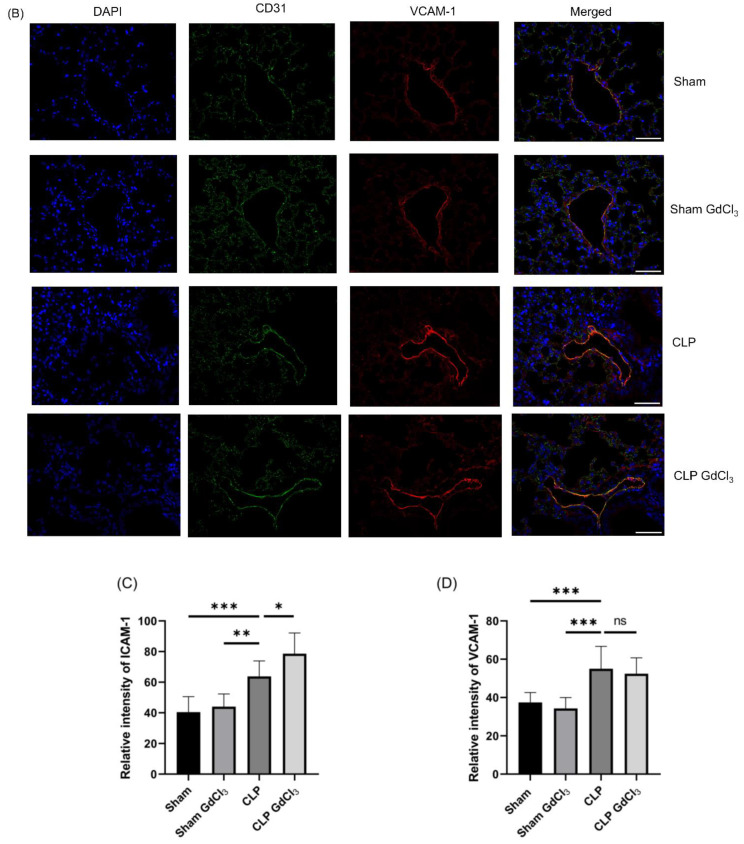
Effect of GdCl_3_ treatment on adhesion molecules immunoreactivity on the pulmonary endothelial cells in sepsis. Scale bar: 50 μm. Representative images of ICAM-1 (**A**) and VCAM-1 (**B**) immunoreactivity (Cy3—red) co-localized with CD-31 (Alexa Fluor 488—green) after 8 h of CLP or sham operation. DAPI staining was used for the visualization of the nucleus. Semi-quantitative analysis of ICAM-1 (**C**) and VCAM-1 (**D**) immunoreactivity on pulmonary endothelial cells was significantly higher in CLP-induced WT mice compared to sham-operated control mice. Data are represented as mean ± SD (*n* = 8). One-way ANOVA with post hoc Tukey test was employed. Statistical significance was determined at * *p* < 0.05; ** *p* < 0.01; *** *p* < 0.001; and ns, not significant.

In mice with CLP-induced sepsis, immunofluorescence microscopy showed higher immunoreactivity of ICAM-1 (Figure 3A–C) and VCAM-1 (Figure 3B–D) that co-localized with structures identified by CD31 staining than in sham-operated control mice. Pretreatment with GdCl_3_ in the CLP-induced septic mice, significantly increased ICAM-1 immunoreactivity in structures localized with CD31 compared to CLP-induced septic mice without GdCl_3_ treatment. However, there was no alteration in the VCAM-1 immunoreactivity in co-localized with CD31 in septic mice with pretreated GdCl_3_ compared to septic mice without GdCl_3_ treatment.

## 4. Discussion

In this study, we investigated the effect of Kupffer cell inactivation on adhesion molecules on endothelial cells following cecal ligation and puncture-induced sepsis in mice. We determined the protein expression of ICAM-1 and VCAM-1 and their immunoreactivity in liver and lung tissues from an experimental CLP sepsis mice model with and without GdCl_3_ pretreatment to compare the effects of GdCl_3_ on these tissues.

In the liver, pretreatment with GdCl_3_ resulted in reduced protein expression of ICAM-1 and its immunoreactivity, which co-localized with LYVE-1 expression in mice with sepsis. The finding that GdCl_3_ reduced the protein expression of ICAM-1 is consistent with previous studies that established the expression of ICAM-1 is mainly regulated by pro-inflammatory cytokines, such as TNF-α. For example, LPS-induced Kupffer cells increased TNF-α production, leading to increased expression of ICAM-1 in liver sinusoidal endothelial cells [22]. Similarly, Kupffer cells from rats with CLP-induced sepsis had a significant increase in the concentration of TNF-α, accompanied by an increase in ICAM-1 mRNA levels in the whole-liver tissue [29]. During cholestatic liver injury, GdCl_3_ treatment reduced the expression of mRNA levels of ICAM-1 in liver tissue [30]. The previous study by our group showed increased TNF-α expression in the liver of CLP-induced septic mice, which was attenuated after GdCl_3_ treatment [23]. Furthermore, GdCl_3_ treatment reduced neutrophil infiltration as accessed by MPO activity assay and liver injury in CLP-induced septic mice. Capillarization/defenestration (decreased diameter, frequency, and porosity) of LSECs is associated with liver injury in sepsis, as shown in animal models and human patients [31]. GdCl_3_ pretreatment in CLP-induced septic mice resulted in significantly reduced defenestration (increased frequency and porosity of LSECs) and fewer gaps compared with mice without GdCl_3_ treatment [23]. The results presented in this study demonstrate that the inactivation of Kupffer cells with GdCl_3_ treatment decreases the protein expression of ICAM-1 in the liver and its immunoreactivity in liver sinusoidal endothelial cells. This suggests that ICAM-1 may be regulated by the production of TNF-α via Kupffer cells. Consequently, ICAM-1 regulated via TNF-α could be a possible cause of the alteration in fenestra numbers and gap formation in LSECs, resulting in liver injury during sepsis.

We also investigated the expression levels of ICAM-1 and VCAM-1 proteins in lung tissue and their immunoreactivity in structures localized with CD31 expression in GdCl_3_-pretreated mice with CLP-induced sepsis. There was a significant elevation in ICAM-1 concentration in the lungs and its immunoreactivity, which co-localized with CD31-expressing structures following GdCl_3_ treatment. This could be attributed to the suppression of endotoxin uptake by Kupffer cells, leading to the overexposure of extrahepatic organs, such as the lungs, to endotoxins. Kupffer cells and pulmonary macrophages exhibit substantial functional differences, indicating heterogeneity between these cells [32]. During sepsis, pulmonary cells (namely pulmonary macrophages) secrete pro-inflammatory cytokines (mainly TNF-α), and it is reasonable to assume that secretion of these mediators increased when removal of bacteria and associated products such as endotoxin by Kupffer cells was inhibited [33,34]. CLP-induced sepsis initiates pulmonary endothelial glycocalyx degradation via TNF-α-dependent mechanisms. Glycocalyx degradation increases the availability of endothelial surface adhesion molecules, contributing to neutrophil adhesion [12]. This degradation of the pulmonary endothelial glycocalyx has been shown to initiate ALI associated with sepsis [12]. A previous study by Gaddam et al. found an increase in TNF-α expression in the lungs in the GdCl_3_-treated group, which may be responsible for the alterations in the expression of ICAM-1 in the lung tissue and pulmonary endothelial cells [23]. This effect may promote the infiltration of neutrophils, ultimately contributing to the onset of lung injury.

Gaddam et al. also demonstrated that GdCl_3_ pretreatment in sepsis increased lung myeloperoxidase [23]. Furthermore, H&E staining of lung sections demonstrated that GdCl_3_ pretreatment failed to decrease the alveolar congestion and leukocyte infiltration caused by sepsis. This could be due to increased neutrophils in the alveoli triggered by cytokine and chemokines released from activated alveolar macrophages. These results indicate that sepsis-induced lung injury is independent of the direct effect of GdCl_3_ on Kupffer cells in the liver [23]. Despite specific inhibition of Kupffer cell activation via GdCl_3_, there is evidence that GdCl_3_ pretreatment affects alveolar macrophage responses to ozone exposure [26]. However, the mechanism of this effect is unclear. As a result, it is possible that GdCl_3_ has independent effects on pulmonary macrophages in CLP-induced septic mice. Similarly, pretreatment with GdCl_3_ protected against lung injury following endotoxemia and sepsis by blocking the uncontrolled release of pro-inflammatory mediators and caspase-3 and elevating IL-10 concentrations released from Kupffer cells. These studies indicated there is a complex relationship between Kupffer cells and pulmonary macrophages in lung injury during endotoxemia and sepsis [27,35,36]. Further investigations are therefore necessary to elucidate the precise role of GdCl_3_ pretreatment in sepsis-induced lung injury.

**Figure 4:**
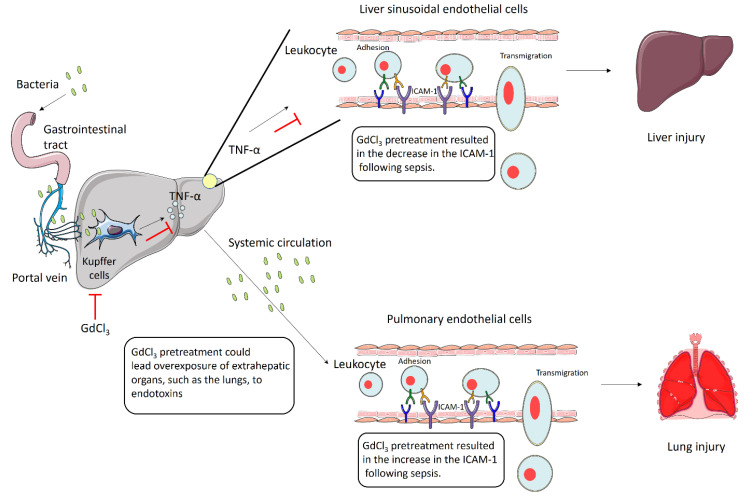
Kupffer cells regulate ICAM-1 in the liver and lungs in CLP-induced sepsis. Inactivation of Kupffer cells when pretreated with GdCl_3_ decreases expression of ICAM-1 on the liver sinusoidal endothelial cells, which may result from a decrease in the release of TNF-α from inactivated Kupffer cells when exposed to endotoxin. However, inactivation with GdCl_3_ pretreatment increases the expression of ICAM-1 on the pulmonary endothelial cells, possibly from overexposure of endotoxin to the lungs. The figure incorporates images from Servier Medical Art, which is licensed under a Creative Commons Attribution 3.0 Unported License (https://creativecommons.org/licenses/by/3.0/, accessed on 1 June 2023).

The results described in the present study suggest that the inactivation of Kupffer cells via GdCl_3_ is responsible for the alteration in ICAM-1 expression in the endothelial cells of the liver and lungs, which regulated the influx of neutrophils and related tissue damage. GdCl_3_ has been found to reduce TNF-α production in the liver [23]. One plausible mechanism by which GdCl_3_ reduces the expression of ICAM-1 is by decreasing locally synthesized TNF-α in the liver. In the lungs, GdCl_3_ treatment may cause overexposure of alveolar macrophages to endotoxin, presumably because of Kupffer cell malfunction, leading to a local increase in TNF-α levels and elevated ICAM-1 expression. The expression of VCAM-1 in the liver and lungs did not change in response to GdCl_3_ exposure (Figure 4).

The structural and functional properties of ICAM-1 and VCAM-1 are similar, and NF-ĸB promotes the expression of both of them. It is intriguing to note that the inactivation of Kupffer via GdCl_3_ has a role in the alteration in the expression of ICAM-1 in the liver and lungs but not that of VCAM-1. In several studies, ICAM-1 and VCAM-1 have been reported to be differentially regulated [37,38,39], but the underlying mechanisms remain unclear. For the first time, our findings indicate that inactivation of Kupffer via GdCl_3_ is one of the differential regulatory factors for ICAM-1 and VCAM-1. The reason that VCAM-1 expression is not affected by GdCl_3_ is not clear. There is a need for further research to examine the complex mechanisms regulating ICAM-1 and VCAM-1 expression.

In conclusion, this study has shown that GdCl_3_ pretreatment in mice with CLP-induced sepsis decreases the protein expression of ICAM-1 in the liver and its immunoreactivity in LSECs but increases ICAM-1 protein expression in the lung and its immunoreactivity in pulmonary endothelial cells compared to CLP-induced mice without GdCl_3_ treatment. However, GdCl_3_ pretreatment had no effect on the expression of VCAM-1 protein in liver and lungs and its immunoreactivity in LSECs and pulmonary endothelial cells. This study enhances our understanding of the role of Kupffer cells, adhesion molecules, and endothelial cells in sepsis and could lead to the development of novel therapeutic approaches for this condition.

## Figures and Tables

**Table 1:** Primary and secondary antibodies used in this study for immunofluorescence microscopy.

Product	Antibody/Type	Source Catalogue No.	Dilution
Immunofluorescence			
ICAM-1	Primary/Goat polyclonal	R&D System, Minneapolis, MN, USA/AF796	1:1000
VCAM-1	Primary/Goat polyclonal	R&D System, Minneapolis, MN, USA/AF643	1:200
LYVE-1	Primary/Rabbit polyclonal	Abcam, Cambridge, UK/ab14917	1:100
CD31	Primary/Rabbit polyclonal	Abcam, Cambridge, UK/ab124432	1:500
Donkey anti-goat	Secondary/Texas Red	Abcam, Cambridge, UK/ab6883	1:1000
Donkey anti-rabbit	Secondary/FITC	Abcam, Cambridge, UK/ab6798	1:1000

## Data Availability

All data generated or analyzed during this study are included in this published article.
